# Key Nutrient Drivers for Biomass and C-Phycocyanin Production in *Spirulina* sp. Revealed by Media Optimization

**DOI:** 10.3390/ijms262110425

**Published:** 2025-10-27

**Authors:** Ivani Nurjannah, Toto Subroto, Ari Hardianto, Lucy Adinisa, Keiichi Mochida

**Affiliations:** 1 Department of Biotechnology, Graduate School, Universitas Padjadjaran, Dipatiukur Campus, Bandung 40132, Indonesia; ivani18001@mail.unpad.ac.id (I.N.); lucy18001@mail.unpad.ac.id (L.A.); 2 Department of Chemistry, Faculty of Mathematics and Natural Science, Universitas Padjadjaran, Jatinangor Campus, Sumedang 45363, Indonesia; a.hardianto@unpad.ac.id; 3 Center for Sustainable Resource Science, RIKEN, Yokohama 230-0045, Japan; keiichi.mochida@riken.jp; 4 RIKEN Baton Zone Program, RIKEN Cluster for Science, Technology and Innovation Hub, Yokohama 230-0045, Japan; 5 Kihara Institute for Biological Research, Yokohama City University, Yokohama 244-0813, Japan; 6 School of Information and Data Sciences, Nagasaki University, Nagasaki 852-8521, Japan

**Keywords:** microalgae, media formulation, nutrient modification, pigment enhancement

## Abstract

Optimizing nutrient formulations is essential to improving the biomass yield and C-phycocyanin (C-PC) productivity of *Spirulina* sp., a cyanobacterium with wide-ranging applications in food, pharmaceutical, and biotechnological industries. This study evaluated the effects of macronutrient modifications on growth and pigment biosynthesis using a two-level full factorial design across eight Zarrouk-based formulations compared to the standard medium. Cultivation experiments were conducted in triplicate, and growth was evaluated using linear growth rate, maximum optical density (OD_680_), and dry biomass, while C-PC was quantified in crude extracts (PCL), dried biomass (PCD), and the purity index (PI). Among the tested formulations, F2 (16 g/L NaHCO_3_, 5 g/L NaNO_3_, 0.25 g/L K_2_HPO_4_) achieved the highest biomass productivity, yielding a 37.6% increase in dry weight and a 38.1% improvement in daily productivity compared to the control. In contrast, F3 (16 g/L NaHCO_3_, 5 g/L NaNO_3_, 1 g/L K_2_HPO_4_) yielded the highest C-PC content, nearly doubling both PCL and PCD values and enhancing pigment purity by 40.2%. ANOVA and interaction analyses confirmed that carbon and nitrogen synergistically promoted biomass formation, while phosphorus had a strong effect on pigment biosynthesis through C:N:P interactions. These findings demonstrate that *Spirulina* sp. requires distinct nutrient balances for optimal growth and pigment formation. Formulation F2 is ideal for maximizing biomass productivity, whereas F3 is optimal for high-value C-PC production. The results provide a rational framework for designing nutrient-efficient cultivation systems to advance sustainable *Spirulina*-based biomanufacturing.

## 1. Introduction

The increasing global demand for natural, sustainable, and bioactive has greatly increased commercial interest in microalgae-based bioproducts. Among various microalgal species, *Spirulina* sp. (also referred to as *Arthrospira*) has emerged as a leading candidate due to its high protein content, rapid growth rate, photosynthetic efficiency, and capacity to produce valuable secondary metabolites such as pigments, vitamins, and polyunsaturated fatty acids [1,2]. Its biomass contains ~60–70% protein (dry weight) and essential amino acids, minerals, and functional pigments, enabling its widespread application in functional food, nutraceutical, and cosmetic industries [2,3].

One of the most prominent pigments derived from *Spirulina* is C-phycocyanin (C-PC), a water-soluble blue phycobiliprotein belonging to the phycobilisome complex, which plays a central role in light-harvesting during photosynthesis [4,5]. Beyond its physiological role, C-PC exhibits strong antioxidant, anti-inflammatory, immunomodulatory, and anticancer properties, making it a highly sought-after compound in pharmaceutical, food-coloring, and biomedical applications [6,7]. Despite its commercial potential, large-scale production of C-PC remains limited by low pigment yields and high production costs [8,9]. Since C-PC is intracellularly accumulated, enhancing its yield requires not only improving pigment biosynthesis but also increasing the overall biomass productivity. Consequently, cultivation strategies that support both high cell density and C-PC accumulation are essential to enhance production efficiency [4,9,10].

Nutrient composition is one of the most influential factors governing microalgal growth and metabolite biosynthesis [11,12]. While several standard media such as Zarrouk and BG-11 have been widely used for cultivating *Spirulina*, these formulations were primarily designed to support general growth and may not be optimized for targeted metabolite production [13,14,15]. In particular, the availability and balance of macronutrients—including carbon (C), nitrogen (N), and phosphorus (P)—are known to significantly influence both biomass formation and secondary metabolite accumulation in microalgae [16]. Carbon functions as the foundational element for all organic molecules and serves as the main energy source driving photosynthesis and biosynthetic pathways related to pigment production, including C-PC [16,17]. Nitrogen plays a central role in protein biosynthesis and is directly involved in the formation of amino acids and phycobiliproteins such as C-PC [9,18]. Phosphorus, on the other hand, is essential for the synthesis of ATP, which supports a wide range of cellular metabolic processes, including those involved in pigment biosynthesis [19,20]. By adjusting the C:N:P ratios in the growth medium, metabolite productivity can be significantly enhanced without the need for complex or costly technologies.

Although the roles of individual nutrients in enhancing pigment production have been studied, the combined effects of multiple nutrients on both biomass and C-PC productivity remain insufficiently characterized [9,12,17,18]. Addressing this gap, the present study investigates the influence of nutrient composition—specifically C, N, and P—on the growth and C-PC production of *Spirulina* sp. Using a factorial experimental design, eight media formulations were developed by modifying the concentrations of key macronutrients, with Zarrouk medium serving as the control. This study aims to identify key nutrient drivers that enhance both biomass accumulation and C-PC yield, thereby contributing to the development of more efficient and economically viable *Spirulina* sp. cultivation systems.

## 2. Results

### 2.1. The Effect of Nutrient Modification on the Growth and Biomass Production of Spirulina

#### 2.1.1. Growth Dynamics of *Spirulina* sp. Under Varying Nutrient Modifications

**Figure 1:**
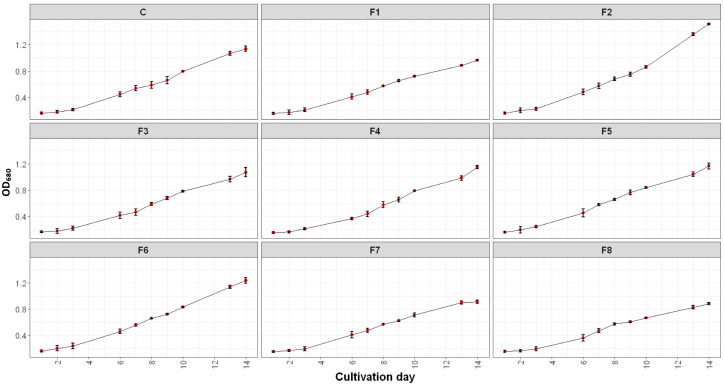
Growth pattern of *Spirulina* sp. under varying nutrient modifications. C: control (Standard Zarrouk medium); F1–F8: Formulation 1–8 (F1: 8 g/L NaHCO_3_, 1.5 g/L NaNO_3_, 0.25 g/L K_2_HPO_4_; F2: 16 g/L NaHCO_3_, 5 g/L NaNO_3_, 0.25 g/L K_2_HPO_4_; F3: 16 g/L NaHCO_3_, 5 g/L NaNO_3_, 1 g/L K_2_HPO_4_; F4: 16 g/L NaHCO_3_, 1.5 g/L NaNO_3_, 0.25 g/L K_2_HPO_4_; F5: 16 g/L NaHCO_3_, 1.5 g/L NaNO_3_, 1 g/L K_2_HPO_4_; F6: 8 g/L NaHCO_3_, 5 g/L NaNO_3_, 0.25 g/L K_2_HPO_4_; F7: 8 g/L NaHCO_3_, 5 g/L NaNO_3_, 1 g/L K_2_HPO_4_; F8: 8 g/L NaHCO_3_, 1.5 g/L NaNO_3_, 1 g/L K_2_HPO_4_). Red dots indicate the average OD_680_ values.

Optical density measurements were conducted to assess the growth dynamics of *Spirulina* sp. and to evaluate the effects of nutrient modifications in the cultivation medium. Optical density was recorded daily at a wavelength of 680 nm over a 14-day cultivation period. The findings revealed distinct growth patterns across different modified media and the control, as illustrated in Figure 1.

Figure 1 shows that all cultures of *Spirulina* sp. exhibited approximately linear increases in OD_680_ throughout the 14-day cultivation period. Each nutrient formulation exerted a distinct effect on the growth trajectory and maximum biomass accumulation. Compared to the control, F1, F7, and F8 showed lower growth rates and reduced final OD_680_ values, suggesting nutrient imbalances that limited proliferation. Conversely, F2 supported the most robust growth, achieving the highest OD_680_ (1.512 ± 0.013) on day 14 and the highest growth rate (0.1030 ± 0.0028 ΔOD/day), representing ~25% higher than the control (0.0820 ± 0.0035 ΔOD/day) (Table 1). These results demonstrate that the nutrient composition of F2 most effectively supported sustained linear growth and biomass accumulation under the tested conditions.

#### 2.1.2. The Effect of Nutrient Modification on *Spirulina* Growth

The effects of carbon, nitrogen, and phosphorus modifications on *Spirulina* sp. growth were systematically assessed using main-effect plots, analysis of variance (ANOVA), and interaction plots. Optical density at 680 nm (OD_680_) was monitored on days 3, 6, 8, 10, 13, and 14, providing a temporal profile of biomass accumulation under different nutrient regimes.

**Figure 2:**
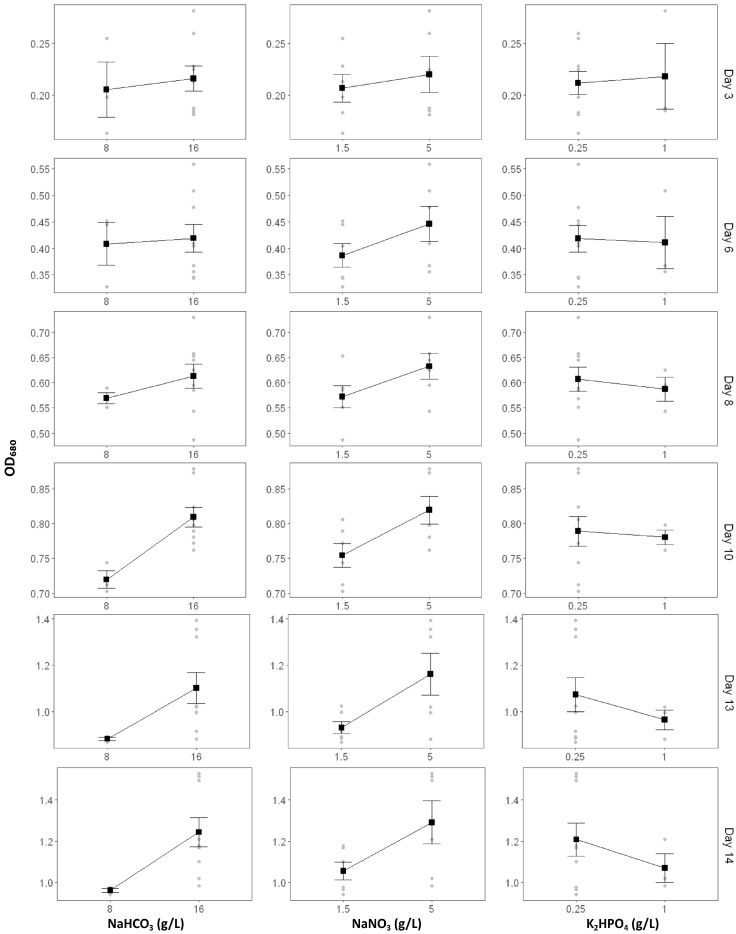
Main effects of carbon, nitrogen, and phosphorus sources on *Spirulina* sp. growth measured as optical density (OD_680_) over time. Grey dots represent individual data points, black squares indicate mean values, and error bars show standard deviations.

The main effect plots (Figure 2) demonstrated that carbon supplementation consistently promoted *Spirulina* sp. growth. Increasing NaHCO_3_ concentration from 8 g/L to 16 g/L resulted in a gradual increase in OD_680_ throughout the cultivation period. Although differences were small and statistically insignificant during the early phase (Day 3–6, *p* > 0.05), the effect became significant on Day 10 (*p* = 2.93 × 10^−2^) and highly significant on Days 13 (*p* = 3.25 × 10^−3^) and 14 (*p* = 1.69 × 10^−5^). This trend highlights the essential role of carbon as an inorganic carbon source and structural substrate for sustained photosynthetic activity, particularly as cell density and metabolic demand increase during the exponential-to-stationary transition.

Nitrogen exerted the strongest and most sustained influence on OD_680_. Raising NaNO_3_ levels from 1.5 g/L to 5 g/L markedly enhanced Spirulina growth after Day 6, with strong statistical significance from Day 8 (*p* = 1.46 × 10^−3^) through Day 14 (*p* = 2.82 × 10^−7^). ANOVA confirmed nitrogen as the key nutrient factor driving biomass accumulation. This is consistent with nitrogen’s central metabolic role in amino acid, pigment, and nucleic acid synthesis, as well as its involvement in chlorophyll formation and photosystem maintenance. This indicates that once carbon is sufficient, nitrogen becomes the primary growth-limiting nutrient.

In contrast, phosphorus exhibited a variable and time-dependent effect. A slight positive response was observed during the early phase (Day 3), but higher phosphorus supplementation (1 g/L K_2_HPO_4_) often resulted in lower OD_680_ during the later stages. Despite this fluctuation, ANOVA revealed that phosphorus became statistically significant from Day 10 (*p* = 3.21 × 10^−2^) and highly significant by Day 13 (*p* = 3.52 × 10^−6^) and Day 14 (*p* = 8.73 × 10^−6^). This indicates that phosphorus acts as a context-dependent regulatory nutrient, influencing energy transfer (ATP, NADPH generation) and facilitating the assimilation of nitrogen and carbon. Rather than being a direct growth driver, phosphorus modulates the metabolic balance and stoichiometric ratios that underpin photosynthetic efficiency and biomass yield.

Collectively, ANOVA (Table 2) confirmed nitrogen as the dominant factor governing *Spirulina* sp. growth, followed by carbon at later stages, with phosphorus exerting modulatory effects. Furthermore, several two-way and three-way interactions were statistically significant during mid-to-late cultivation, underscoring that *Spirulina* sp. growth depends not only on absolute nutrient concentrations but also on the proportional nutrient balance and their synergistic interactions.

**Figure 3:**
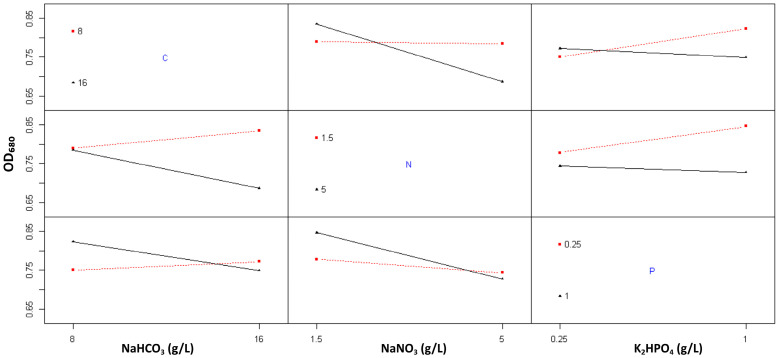
Interaction plots of nutrient effects on the optical density of *Spirulina* sp. Red squares represent the lower factor levels, black triangles represent the higher factor levels, and blue letters indicate the corresponding nutrient components.

The interaction plot matrix (Figure 3) provides a comprehensive visualization of the multivariate effects of carbon, nitrogen, and phosphorus concentrations on *Spirulina* sp. growth, as reflected by OD_680_ values. These graphical trends corroborate the ANOVA results and elucidate the complexity of nutrient interplay underlying cellular growth regulation. The presence of crossing interaction patterns across all nutrient pairs (C:N, C:P, and N:P) indicates that *Spirulina* sp. growth is predominantly governed by multi-nutrient synergies rather than by single-factor effects.

The interaction between carbon and nitrogen revealed a downward trend in OD_680_ with increasing nitrogen levels, particularly under high carbon availability (16 g/L). This pattern suggests that excess nitrogen may trigger a metabolic shift that diverts reducing power and ATP from carbon fixation pathways toward nitrogen assimilation, thereby moderating overall biomass accumulation. Conversely, at lower carbon concentrations (8 g/L), this inhibitory effect diminishes, implying that limited carbon supply constrains nitrogen utilization efficiency. This trend is consistent with the ANOVA findings, which identified the C:N term as highly significant (*p* < 0.01), confirming that the response of Spirulina to nitrogen enrichment is conditional upon carbon availability.

The C:P interaction exhibited nearly parallel trajectories with a subtle upward gradient, indicating that phosphorus supplementation slightly enhanced OD_680_ at both carbon levels. Although the improvement was modest, this trend underscores phosphorus’s role as an auxiliary nutrient that stabilizes cellular energetics and facilitates carbon assimilation. Phosphorus enrichment likely sustains photosynthetic performance by supporting nucleotide synthesis and energy transfer reactions, particularly under carbon-sufficient conditions. The consistency of this interaction pattern highlights that adequate phosphorus availability is indispensable for maintaining metabolic homeostasis and preventing energy limitation during exponential growth.

A clear interdependence between nitrogen and phosphorus availability was also evident from the N:P interaction plot. At low phosphorus levels (0.25 g/L), nitrogen enrichment had negligible or even adverse effects on OD_680_, suggesting that phosphorus scarcity impairs nitrogen assimilation capacity. In contrast, under high phosphorus conditions (1 g/L), nitrogen addition markedly enhanced growth, indicating that phosphorus availability regulates the efficiency of nitrogen metabolism [21]. Statistically, this interaction showed strong significance (*p* < 0.01), demonstrating that the stoichiometric balance between nitrogen and phosphorus is a critical determinant of *Spirulina*’s growth performance.

#### 2.1.3. The Effect of Nutrient Modification on Biomass Production

**Figure 4:**
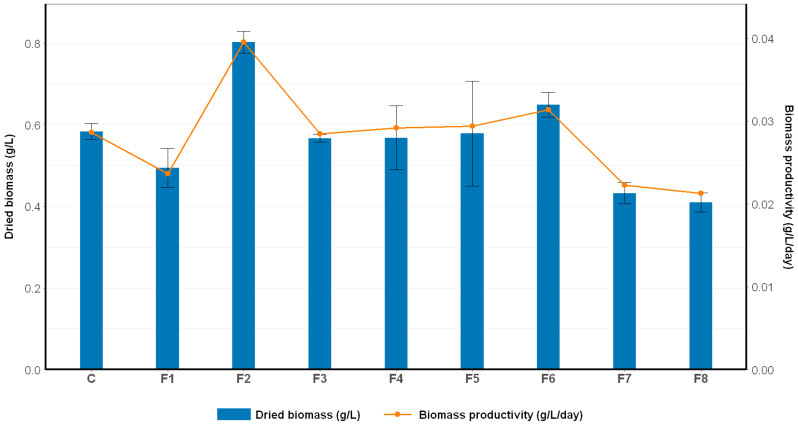
Dried biomass yield (blue) and daily biomass productivity (orange) of *Spirulina* sp. under different nutrient formulations. Values represent mean ± SD from three independent replicates. (C: control (Standard Zarrouk medium); F1–F8: Formulation 1–8 (F1: 8 g/L NaHCO_3_, 1.5 g/L NaNO_3_, 0.25 g/L K_2_HPO_4_; F2: 16 g/L NaHCO_3_, 5 g/L NaNO_3_, 0.25 g/L K_2_HPO_4_; F3: 16 g/L NaHCO_3_, 5 g/L NaNO_3_, 1 g/L K_2_HPO_4_; F4: 16 g/L NaHCO_3_, 1.5 g/L NaNO_3_, 0.25 g/L K_2_HPO_4_; F5: 16 g/L NaHCO_3_, 1.5 g/L NaNO_3_, 1 g/L K_2_HPO_4_; F6: 8 g/L NaHCO_3_, 5 g/L NaNO_3_, 0.25 g/L K_2_HPO_4_; F7: 8 g/L NaHCO_3_, 5 g/L NaNO_3_, 1 g/L K_2_HPO_4_; F8: 8 g/L NaHCO_3_, 1.5 g/L NaNO_3_, 1 g/L K_2_HPO_4_).

The total dry biomass at the end of the cultivation period and the daily biomass productivity were evaluated to assess the impact of nutrient modification on the biomass production of *Spirulina* sp. As shown in Figure 4, significant variations in biomass yield were observed across the modified media formulations (F1–F8) compared to the control. Among all treatments, F2 exhibited the highest performance, achieving a dried biomass of 0.8031 ± 0.0266 g/L, which represents a 37.6% increase compared to the control (0.5836 ± 0.0194 g/L). Similarly, biomass productivity reached 0.0395 ± 0.0011 g/L/day, corresponding to a 38.1% improvement over the control (0.0286 ± 0.0027 g/L/day). These results indicate that the nutrient composition of F2 offered a more favorable stoichiometric balance, enhancing efficient carbon assimilation and increasing metabolic flux toward biomass synthesis.

F2 contained 16 g/L of NaHCO_3_, a carbon source concentration nearly equivalent to that of the control (16.8 g/L), ensuring sufficient carbon availability to support photosynthetic processes. Additionally, the significant increase in nitrogen supply—through 5 g/L of NaNO_3_ compared to 1.5 g/L in the control—offered ample nitrogen to sustain the biosynthesis of proteins, enzymes, and other nitrogenous metabolites. Although the phosphorus concentration in F2 (0.25 g/L K_2_HPO_4_) was lower than in the control (0.5 g/L), the optimized carbon and nitrogen availability compensated for this, allowing the cells to maintain nutrient balance, enhance metabolic efficiency, and sustain robust growth.

In contrast, treatments F7 and F8 yielded the lowest biomass dried weight (0.4323 ± 0.0268 and 0.4100 ± 0.0236 g/L, respectively) and correspondingly reduced productivity values (0.0222 ± 0.0007 and 0.0212 ± 0.0004 g/L/day). These results reflect a clear nutrient imbalance, particularly with regard to carbon availability (as indicated by low biomass yields), which was the primary limiting factor. Both formulations contained only 8 g/L NaHCO_3_—substantially lower than the control (16.8 g/L) and F2 (16 g/L)—thereby severely limiting the carbon supply required for photosynthesis and primary metabolite synthesis. While the nitrogen content in F7 (5 g/L NaNO_3_) was identical to that in F2, the carbon deficiency could not be compensated, resulting in suppressed growth and reduced biomass productivity. Moreover, the elevated phosphorus concentration (1 g/L K_2_HPO_4_) in F7 and F8, in the absence of adequate carbon, may have disrupted the cellular nutrient balance and further impaired the metabolic efficiency of *Spirulina* sp. Consequently, both formulations led to significantly lower biomass production compared to both the control and F2.

#### 2.1.4. The Effect of Nutrient Modification on C-PC Production

C-PC content in *Spirulina* sp. biomass was quantified spectrophotometrically using the method of Bennett and Bogorad (1978), which enables quantification of C-PC in both crude extracts (phycocyanin content in liquid, PCL) and dried biomass (phycocyanin content in dried biomass, PCD) [22].

**Figure 5:**
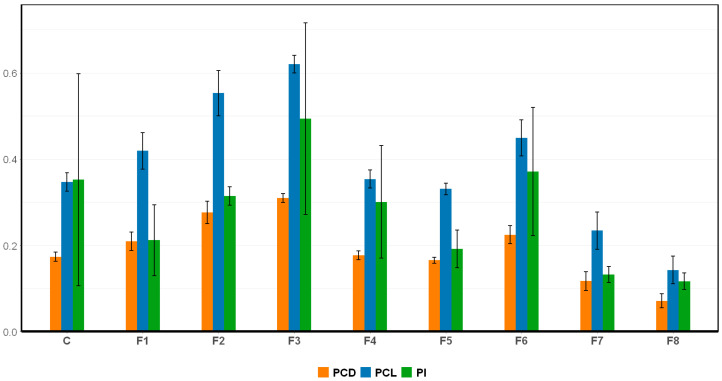
C-PC content under different nutrient modification treatments. (PCL: phycocyanin content in liquid, mg/mL; PCD: phycocyanin content in dried biomass, mg/mg; and PI: purity index; C: control (Standard Zarrouk medium); F1–F8: Formulation 1–8 (F1: 8 g/L NaHCO_3_, 1.5 g/L NaNO_3_, 0.25 g/L K_2_HPO_4_; F2: 16 g/L NaHCO_3_, 5 g/L NaNO_3_, 0.25 g/L K_2_HPO_4_; F3: 16 g/L NaHCO_3_, 5 g/L NaNO_3_, 1 g/L K_2_HPO_4_; F4: 16 g/L NaHCO_3_, 1.5 g/L NaNO_3_, 0.25 g/L K_2_HPO_4_; F5: 16 g/L NaHCO_3_, 1.5 g/L NaNO_3_, 1 g/L K_2_HPO_4_; F6: 8 g/L NaHCO_3_, 5 g/L NaNO_3_, 0.25 g/L K_2_HPO_4_; F7: 8 g/L NaHCO_3_, 5 g/L NaNO_3_, 1 g/L K_2_HPO_4_; F8: 8 g/L NaHCO_3_, 1.5 g/L NaNO_3_, 1 g/L K_2_HPO_4_).

As presented in Figure 5, PCL, PCD, and PI were evaluated across different nutrient modification treatments. Substantial variations were observed among treatments, indicating that nutrient composition strongly influenced both the yield and quality of C-phycocyanin (C-PC). Among all formulations, F3 and F2 exhibited superior performance, yielding the highest C-PC concentrations and purity compared to the control. Specifically, F3 recorded the highest PCL (0.6200 ± 0.0204 mg/mL) and PCD (0.3100 ± 0.0102 mg/mL) values, accompanied by the highest PI of 0.4936 ± 0.2222, which represents an approximately 40.9% improvement over the control (PI = 0.3522 ± 0.2459). Likewise, F2 achieved PCL and PCD values of 0.5526 ± 0.0524 mg/mL and 0.2763 ± 0.0262 mg/mg, respectively—significantly exceeding those of the control (0.3469 ± 0.0213 mg/mL PCL and 0.1734 ± 0.0106 mg/mL PCD). These results demonstrate that both F2 and F3 formulations effectively enhanced phycocyanin biosynthesis, likely by optimizing nitrogen and phosphorus availability and thereby promoting protein assembly and pigment stability.

In contrast, F4 and F5—which contained the same nitrogen concentration as the control (1.5 g/L NaNO_3_)—produced lower C-PC levels, ranging from 0.3307 ± 0.0137 to 0.3537 ± 0.0212 mg/mL PCL and 0.1653 ± 0.0067 to 0.1768 ± 0.0106 mg/mg PCD, highlighting nitrogen availability as a primary limiting factor for C-PC biosynthesis. Furthermore, a clear positive correlation was observed between high PCL and PCD values and increased PI. F3, which showed the highest C-PC concentration, also recorded the highest PI at 0.4936 ± 0.2222—a 40.23% improvement over the control. Conversely, F7 and F8, which showed the lowest PCL and PCD values, also exhibited the lowest PI values, at 0.1322 ± 0.0187 and 0.1166 ± 0.0191, respectively.

**Figure 6:**
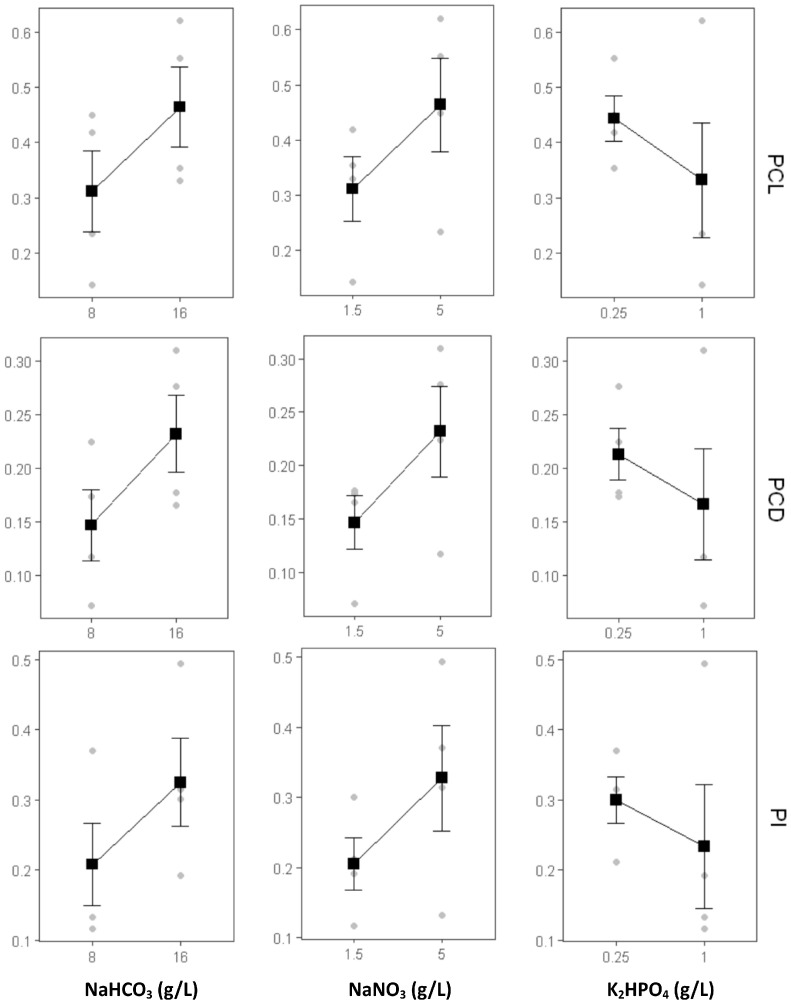
Main effects of carbon, nitrogen, and phosphorus sources on PCL (phycocyanin content in liquid), PCD (phycocyanin content in dried biomass), and PI (purity index). Grey dots represent individual data points, black squares indicate mean values, and error bars show standard deviations.

**Figure 7:**
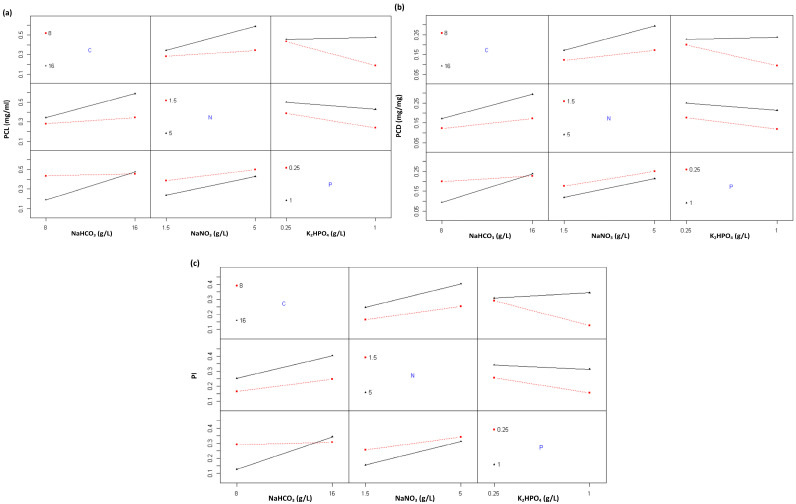
Interaction plots of nutrient concentrations on C-PC responses in *Spirulina* sp. (**a**) PCL (mg mL^−1^), (**b**) PCD (mg mg^−1^), and (**c**) PI (Purity Index). Red squares represent the lower factor levels, black triangles represent the higher factor levels, and blue letters indicate the corresponding nutrient components.

To further examine the contribution of individual nutrient components—including carbon (NaHCO_3_), nitrogen (NaNO_3_), and phosphorus (K_2_HPO_4_)—and their interactions, statistical analyses were conducted. These included main effect plots (Figure 6), interaction plots (Figure 7), and ANOVA (Table 3).

Increasing the carbon concentration from 8 g/L to 16 g/L had a statistically significant positive effect on both the quantity (PCL and PCD) and quality (PI) of C-PC. The upward trend observed in the main effect plot (Figure 6) indicates that carbon, as a primary energy source, plays a key role in supporting the metabolic pathways involved in C-PC biosynthesis. Higher carbon availability is known to enhance pigment synthesis by stimulating both primary and secondary metabolic activity [23,24]. These findings are supported by ANOVA results (Table 3), where carbon showed a highly significant effect (*p* = 9.60 × 10^−9^). Similarly, increasing the nitrogen concentration from 1.5 g/L to 5 g/L NaNO_3_ also significantly boosted C-PC production, as shown by the ascending trend in the main effect plot. ANOVA confirmed the significant influence of nitrogen (*p* = 2.60 × 10^−9^), reinforcing its central role in protein biosynthesis, including phycobiliproteins such as C-PC.

On the other hand, increasing the phosphorus concentration from 0.25 g/L to 1 g/L showed a negative trend across PCL, PCD, and PI, as indicated by the downward-sloping curve in the main effect plot. Although F3—containing 1 g/L K_2_HPO_4_—produced the highest C-PC content, the main effect plot reflects the overall trend across all treatments. These findings suggest that elevated phosphorus concentrations may inhibit C-PC biosynthesis unless accompanied by optimized carbon and nitrogen levels, as in F3. This inverse relationship was statistically significant, with ANOVA indicating a strong negative effect of phosphorus on C-PC production (*p* = 2.79 × 10^−7^).

The interaction plots (Figure 7) provide additional insight into the synergistic or antagonistic relationships among nutrients influencing C-PC synthesis. The carbon–nitrogen interaction demonstrated a clear synergistic effect. At high nitrogen levels (5 g/L NaNO_3_), increasing carbon concentration (from 8 to 16 g/L NaHCO_3_) substantially improved PCL, PCD, and PI values (black line). Conversely, under low-nitrogen conditions (1.5 g/L NaNO_3_), carbon enrichment produced only a marginal response (flat red line). This interaction was statistically significant (*p* = 4.00 × 10^−6^; Table 3), emphasizing that simultaneous optimization of carbon and nitrogen availability is essential for maximizing C-PC biosynthesis in *Spirulina* sp.

The carbon–phosphorus interaction displayed a similar but more nuanced pattern. When phosphorus concentration was high (1 g/L K_2_HPO_4_), increasing carbon markedly enhanced C-PC production (steep black line), indicating a strong synergistic response. Under low phosphorus conditions (0.25 g/L K_2_HPO_4_); however, carbon enrichment had little effect (flat red line). Notably, C-PC levels sharply declined when phosphorus increased under carbon-limiting conditions, suggesting that phosphorus can only stimulate pigment biosynthesis when both carbon (16 g/L NaHCO_3_) and nitrogen (5 g/L NaNO_3_) are sufficiently supplied. This indicates a C:N-dependent role for phosphorus in the pigment assembly process. The interaction was highly significant (*p* = 1.95 × 10^−8^; Table 3).

The nitrogen–phosphorus interaction revealed a more complex, partially antagonistic relationship. Enhanced C-PC production occurred with nitrogen enrichment under low phosphorus conditions but further increases in phosphorus consistently reduced pigment quantity and purity across nitrogen levels. This interaction was statistically significant (*p* = 1.47 × 10^−2^), indicating that phosphorus excess acts as a suppressive factor in C-PC biosynthesis. These results suggest that a moderate phosphorus supply (0.25 g/L K_2_HPO_4_) is sufficient to fulfill cellular demand for pigment formation in *Spirulina* sp., while higher concentrations may disrupt metabolic balance or induce oxidative stress, leading to pigment degradation [25].

## 3. Discussion

The findings of this study demonstrate that nutrient composition—particularly the availability of inorganic carbon, nitrogen, and phosphorus—critically influences both biomass productivity and C-PC biosynthesis in *Spirulina* sp. Among the eight tested formulations, F2 and F3 consistently outperformed the control (standard Zarrouk medium) in terms of cell growth, biomass accumulation, and pigment production, underscoring the importance of nutrient stoichiometry in optimizing Spirulina metabolism.

Specifically, formulation F2, which contained 16 g/L NaHCO_3_, 5 g/L NaNO_3_, and 0.25 g/L K_2_HPO_4_, supported the most robust growth and biomass formation throughout the 14-day cultivation period. The culture reached the highest optical density (OD_680_ = 1.512 ± 0.013) and exhibited a growth rate of 0.1030 ± 0.0028 ΔOD/day, representing an approximately 25% increase over the control (0.0820 ± 0.0035 ΔOD/day). Correspondingly, F2 achieved a dry biomass concentration of 0.8031 ± 0.0266 g/L and a biomass productivity of 0.0395 ± 0.0011 g/L/day, which were 37.6% and 38.1% higher, respectively, than those of the control (0.5836 ± 0.0194 g/L and 0.0286 ± 0.0027 g/L/day). This improvement is attributed to the synergistic effect of high concentrations of carbon and nitrogen, which together create a nutrient-rich environment that accelerates cellular proliferation.

Inorganic carbon derived from NaHCO_3_ serves as the primary substrate in the Calvin cycle, where carbon dioxide is fixed by the enzyme RuBisCO into 3-phosphoglycerate (3-PGA), which is subsequently converted into glyceraldehyde-3-phosphate (GAP). GAP then enters key metabolic pathways such as glycolysis and the TCA cycle, generating essential precursors like acetyl-CoA and α-ketoglutarate, which are substrates for the biosynthesis of aminolevulinic acid (ALA)—the initial precursor in the tetrapyrrole biosynthetic pathway [23,26]. This pathway is essential for the synthesis of phycocyanobilin (PCB), the chromophore that imparts optical and bioactive properties to C-PC [24].

Concurrently, nitrogen supplied through NaNO_3_ is indispensable for protein biosynthesis, including that of the C-PC apoprotein, as well as for the formation of ALA via both the C5 (glutamate-based) and C4 (glycine and succinyl-CoA) pathways. ALA is further metabolized into uroporphyrinogen III, which subsequently gives rise to heme and, eventually, PCB via the sequential actions of heme oxygenase and PcyA enzymes [18]. The conjugation of PCB with the apoprotein results in the functional holoprotein C-PC, which is structurally integrated into the phycobilisome, the primary light-harvesting antenna complex in cyanobacteria and microalgae [9,26].

While F2 was optimal for biomass production, F3 (1 g/L K_2_HPO_4_, 5 g/L NaNO_3_, and 16 g/L NaHCO_3_) exhibited the highest C-PC productivity, yielding PCL values of 0.6200 ± 0.0204 mg/mL and PCD of 0.3100 ± 0.0102 mg/mL—nearly twice those obtained in the control medium (PCL = 0.3469 ± 0.0213 mg/mL; PCD = 0.1734 ± 0.0106 mg/mL). These results suggest that elevated phosphate concentrations, when accompanied by sufficient levels of nitrogen and carbon, can effectively enhance C-PC biosynthesis. Phosphate plays a key role in cellular energy metabolism, particularly in the regeneration of ATP and NADPH, which are required in reductive biosynthesis and secondary metabolite production such as pigment formation [19,20].

Furthermore, sufficient phosphate levels may enhance enzyme activities throughout the biosynthetic cascade, from ALA and porphyrin synthesis to apoprotein translation. Phosphate is also indirectly involved in protein biosynthesis regulation by modulating translational pathways and stabilizing RNA structures [19,27]. In F3, the increased ATP and NADPH availability may have supported efficient mRNA translation of the C-PC apoprotein and post-translational modifications essential for pigment–protein complex formation. Nevertheless, excess phosphate in the absence of adequate macronutrients may lead to metabolic imbalances, phosphate accumulation, and oxidative stress [28]. Therefore, the optimal performance of F3 confirms that elevated phosphate levels are only beneficial for C-PC biosynthesis when adequate nitrogen and carbon are concurrently available to support the complete biosynthetic machinery.

Interestingly, despite having a lower phosphate concentration (0.25 g/L), F2 also exhibited a significant increase in C-PC production (PCL = 0.5526 ± 0.0524 mg/mL; PCD = 0.2763 ± 0.0262 mg/mg). This suggests that adequate availability of carbon and nitrogen alone may still drive efficient pigment production, even under moderate phosphate conditions. In contrast, formulations F4 and F5, which had variable phosphate levels but reduced nitrogen concentrations (3 g/L and 1.5 g/L NaNO_3_, respectively), did not show notable improvements in pigment synthesis, indicating that nitrogen was the primary limiting factor in C-PC biosynthesis under these conditions. ANOVA further confirmed that the interactive effects of carbon, nitrogen, and phosphate were significant (*p* < 0.05) in influencing C-PC production. This reinforces the notion that nutrient balance, rather than individual nutrient concentration, is crucial for optimal pigment biosynthesis.

Micronutrients in formulations F2 and F3 were deliberately reduced by 50% relative to the standard Zarrouk medium, following the approach of Zuorro et al. (2021), who demonstrated that *Oscillatoria* sp. exhibited no growth deficit when micronutrient concentrations were halved [29]. Our results corroborate this finding for *Spirulina* sp., showing that growth and pigment production remained unaffected. This confirms the species’ remarkable metabolic flexibility and adaptability when major macronutrients are adequately supplied. Such nutrient efficiency offers practical benefits for large-scale cultivation by lowering medium costs without compromising productivity.

The findings of this study confirm that integrated optimization of carbon, nitrogen, and phosphate levels substantially enhances C-PC productivity and quality. Formulations F2 and F3 offer sustainable and economically viable strategies for cultivating *Spirulina* sp., applicable to biomass and high-value pigment production. These findings open avenues for designing media tailored to the specific metabolic needs of cyanobacteria, with promising applications in functional food, cosmetics, and industrial biotechnology.

## 4. Materials and Methods

### 4.1. Experimental Design

This study employed a 2-level full factorial experimental design using the FrF2 package in R version 4.2.2 (R Core Team, 2022) through Jupyter Notebook to investigate the effects of carbon, nitrogen, and phosphorus sources on the growth rate, biomass productivity, and C-PC production of *Spirulina* sp. The tested variables included concentrations of NaHCO_3_, NaNO_3_, and K_2_HPO_4_ at two levels: 8 and 16 g/L, 1.5 and 5 g/L, and 0.25 and 1 g/L, respectively. The selected concentration ranges were determined based on previous studies reporting that moderate to high bicarbonate and nitrogen levels enhance *Spirulina* growth and phycocyanin synthesis, while excessive nutrient levels (>16 g/L NaHCO_3_ and >4.5 g/L NaNO_3_) may induce osmotic or pH stress [9,17,27]. Similarly, the phosphorus levels were chosen to represent low and high concentrations that support optimal pigment accumulation and metabolic activity [30,31]. Eight experimental runs were conducted based on the matrix shown in Table 4 over 14 days. The response variables measured at the end of cultivation included OD_680_, dried biomass, and both the quantity and quality of C-PC produced. A standard Zarrouk medium served as the control.

Experimental results were visually analyzed using main effect and interaction plots generated via the FrF2 and ggplot2 packages, to observe trends in the individual and interactive effects of the variables on the responses. In addition, Analysis of Variance (ANOVA) was conducted to assess the statistical significance of each factor and their interactions at a 95% confidence level [32,33].

### 4.2. Culture and Cultivation Conditions

The microalgae used in this study was a fresh culture of *Spirulina* sp. obtained from Balai Perikanan Budidaya Air Payau (BPBAP) Situbondo, Jawa Timur, Indonesia. The culture was transferred into sterilized Zarrouk medium and cultivated at 25 °C under LED lamps (4000 K) HiLED (Jakarta, Indonesia). The standard Zarrouk medium contains 16.8 g/L NaHCO_3_, 1.5 g/L NaNO_3_, and 0.5 g/L K_2_HPO_4_ as the main sources of carbon, nitrogen, and phosphorus, respectively. These macronutrients are optimized for general *Spirulina* growth but not specifically for targeted metabolite production [14,34]. The complete formulation, including micronutrients and trace elements, is provided in Table 5. During the experimental cultivation, microalgal cultures were grown in eight different nutrient compositions (F1–F8) as detailed in Table 5. Micronutrient and trace element concentrations were reduced to 50% of those in the standard Zarrouk formulation [28,35]. The NaCl concentration was maintained at 1 g/L, identical to the standard medium, as this salinity level is optimal for *Spirulina* sp. growth [36].

Cultures were grown in 500 mL photobioreactors containing 250 mL of sterilized nutrient medium. Cultivation was carried out for 14 days at 25 °C with continuous agitation at 100 rpm to ensure uniform mixing and prevent cell sedimentation. Illumination was provided by white LED lamps (4000 K) at an intensity of 5000 lux, measured at the culture surface using a lux meter, with a 12:12 h light–dark photoperiod. Optical density at 680 nm (OD_680_) was measured daily in three independent biological replicates to monitor growth [32]. Light intensity was not adjusted during the cultivation period; consequently, Once OD_680_ exceeded ~0.1, cultures became light-limited and growth was approximately linear rather than exponential. The growth rate was therefore determined as the slope of OD_680_ versus cultivation time (ΔOD/day) using linear regression over the 14-day period. Growth rates from three biological replicates were averaged and expressed as mean ± standard deviation.

### 4.3. Dried Biomass and Productivity Analysis

*Spirulina* sp. biomass was harvested by filtration, washed with distilled water to remove surface-bound inorganic residues, and dried at 60 °C to a constant weight. The dried biomass and productivity were determined using the following equations [37,38]:

$$\mathrm{Dried}\;\mathrm{biomass}\;(g/L)\;=\;\frac{{(m}_{biomass+filter})-(m_{filter})\;(g)}{Volume(L)}$$

$$\mathrm{Biomass}\;\mathrm{productivity}\;(g/L/\mathrm{day})=\frac{dried\;biomass\;(g/L)}{cultivation\;day\;(day)}$$

Daily biomass values were estimated using a regression equation derived from the calibration curve of optical density versus dried biomass (see Appendix A).

### 4.4. C-Phycocyanin (C-PC) Content Analysis

C-PC content was analyzed spectrophotometrically, following the method described by Bennett & Bogorad (1978) [22]. Extraction was performed using a freeze–thaw technique involving cycles of freezing and thawing dry *Spirulina* biomass. Specifically, 100 mg of dry biomass was suspended in 10 mL of 0.2 M phosphate buffer at pH 7. The sample was homogenized and stored at −4 °C for 24 h, then incubated at room temperature for 20 min. This cycle was repeated twice to maximize extraction efficiency. The suspension was then centrifuged, and the blue-colored supernatant was collected for analysis [39,40].

Absorbance measurements were taken using a UV-Vis spectrophotometer at 615 nm, 652 nm, and 280 nm. The C-PC content in the liquid extract (PCL), C-PC content in the dried biomass (PCD), and purity index (PI) were calculated using the following equations [22]:

$$\mathrm{PCL}\;(\mathrm{mg}/\mathrm{mL})\;=\;\frac{A_{615}-0.474{(A}_{652})}{5.34}$$

$$\mathrm{PCD}\;(\mathrm{mg}/\mathrm{mg})=\frac{\left[PCL\right]\times extract\;volume\;(mL)}{biomass\;(mg)}$$

$$\mathrm{PI}=\frac{A_{615}}{A_{280}}$$

## 5. Conclusions

This study demonstrates that optimizing the stoichiometric balance of inorganic carbon, nitrogen, and phosphorus significantly enhances both biomass productivity and C-PC biosynthesis in *Spirulina* sp. Among the tested formulations, F2 (16 g/L NaHCO_3_, 5 g/L NaNO_3_, 0.25 g/L K_2_HPO_4_) achieved the highest biomass productivity, yielding a 37.6% increase in dry weight and a 38.1% improvement in daily productivity compared to the control. In contrast, F3 (16 g/L NaHCO_3_, 5 g/L NaNO_3_, 1 g/L K_2_HPO_4_) produced the highest C-PC content, with PCL and PCD values of 0.6200 mg/mL and 0.3100 mg/mL, respectively—nearly double those of the control—and enhanced pigment purity by 40.2%. Importantly, the C:N:P ratios for these optimized formulations differed substantially: F2 exhibited a ratio of approximately 160:50:2.5, favoring cell growth and biomass accumulation, whereas F3 showed a ratio of approximately 160:50:10, promoting enhanced pigment synthesis. These quantitative outcomes confirm that *Spirulina* sp. growth and pigment production are governed by nutrient stoichiometry rather than single-nutrient enrichment. These optimized nutrient ratios improved photosynthetic efficiency, energy metabolism, and pigment assembly, providing a framework for nutrient-efficient Spirulina cultivation.

## Figures and Tables

**Table 1:** Growth rate and maximum OD_680_ of *Spirulina* sp. under different nutrient formulations.

Formulation	Growth Rate (ΔOD/Day)	OD_680_ Max
Control	0.0820 ± 0.0035	1.136 ± 0.070
F1	0.0654 ± 0.0030	0.963 ± 0.017
F2	0.1030 ± 0.0028	1.512 ± 0.013
F3	0.0727 ± 0.0032	1.070 ± 0.099
F4	0.0775 ± 0.0008	1.149 ± 0.033
F5	0.0792 ± 0.0060	1.164 ± 0.062
F6	0.0901 ± 0.0040	1.233 ± 0.073
F7	0.0612 ± 0.0050	0.913 ± 0.042
F8	0.0599 ± 0.0045	0.983 ± 0.164

**Table 2:** ANOVA summary of carbon, nitrogen, phosphorus sources, and their interactions on the optical density (OD_680_) of *Spirulina* sp. across cultivation days.

	*p*-Value					
	Day 3	Day 6	Day 8	Day 10	Day 13	Day 14
C	9.09 × 10^−1^	9.50 × 10^−1^	4.93 × 10^−1^	2.93 × 10^−2^ *	3.25 × 10^−3^ **	1.69 × 10^−5^ ***
N	2.77 × 10^−1^	5.81 × 10^−2^	1.46 × 10^−3^ **	2.86 × 10^−6^ ***	1.79 × 10^−8^ ***	2.82 × 10^−7^ ***
P	9.28 × 10^−1^	9.13 × 10^−1^	1.57 × 10^−1^	3.21 × 10^−2^ *	3.52 × 10^−6^ ***	8.73 × 10^−6^ ***
C:N	2.91 × 10^−1^	8.23 × 10^−1^	2.11 × 10^−1^	5.97 × 10^−6^ ***	1.28 × 10^−2^ *	4.47 × 10^−4^ ***
C:P	9.41 × 10^−1^	5.94 × 10^−1^	1.83 × 10^−1^	4.23 × 10^−4^ ***	1.44 × 10^−5^ ***	4.26 × 10^−5^ ***
N:P	8.01 × 10^−1^	1.76 × 10^−1^	9.03 × 10^−2^	1.38 × 10^−3^ **	5.63 × 10^−8^ ***	4.36 × 10^−6^ ***
C:N:P	6.90 × 10^−1^	6.41 × 10^−1^	8.55 × 10^−2^	5.02 × 10^−2^ *	2.83 × 10^−4^ ***	2.56 × 10^−4^ ***

Significance codes: * *p <* 0.05; ** *p <* 0.01; *** *p <* 0.001. ANOVA revealed that carbon effects became significant from Day 10 onward, nitrogen showed strong significance starting Day 8, while phosphorus displayed context-dependent effects at later stages. Several interaction terms (C:N, C:P, N:P, C:N:P) were also significant after Day 10, reflecting the importance of nutrient balance at higher cell densities.

**Table 3:** ANOVA summary of carbon, nitrogen, phosphorus, and their interactions on C-phycocyanin (C-PC) production of *Spirulina* sp.

	Df	Sum Sq	Mean Sq	F Value	*p*-Value	
C	1	363.0	363.0	122.1	9.60 × 10^−9^	***
N	1	348.5	348.5	117.2	2.60 × 10^−9^	***
P	1	186.9	186.9	62.8	2.79 × 10^−7^	***
C:N	1	126.2	126.2	42.4	4.00 × 10^−6^	***
C:P	1	268.6	268.6	90.3	1.95 × 10^−8^	***
N:P	1	21.6	21.6	7.28	1.47 × 10^−2^	*
C:N:P	1	0.8	0.8	0.26	6.15 × 10^−1^	
Residuals	18	53.3	2.97			

Significance codes: * *p <* 0.05; *** *p* < 0.001. C-PC production was significantly influenced by carbon, nitrogen, and phosphorus (*p* < 0.001), with strong two-way interactions (C:N, C:P, N:P). The three-way interaction (C:N:P) was not significant, indicating that pigment biosynthesis was mainly regulated by individual nutrients and pairwise interactions rather than full-factor synergy.

**Table 4:** 2-level full factorial experimental design.

Run	NaHCO_3_ (g/L)	NaNO_3_ (g/L)	K_2_HPO_4_ (g/L)
F1	8	1.5	0.25
F2	16	5	0.25
F3	16	5	1
F4	16	1.5	0.25
F5	16	1.5	1
F6	8	5	0.25
F7	8	5	1
F8	8	1.5	1

**Table 5:** Nutrient composition of the growth medium for *Spirulina* sp.

Component	Zarrouk (g/L)	F1 (g/L)	F2 (g/L)	F3 (g/L)	F4 (g/L)	F5 (g/L)	F6 (g/L)	F7 (g/L)	F8 (g/L)
NaHCO_3_	16.8	8	16	16	16	16	8	8	8
NaNO_3_	1.5	1.5	5	5	1.5	1.5	5	5	1.5
K_2_HPO_4_	0.5	0.25	0.25	1	0.25	1	0.25	1	1
NaCl	1	1	1	1	1	1	1	1	1
CaCl_2_.2H_2_O	0.04	0.02	0.02	0.02	0.02	0.02	0.02	0.02	0.02
FeSO_4_.7H_2_O	0.01	0.005	0.005	0.005	0.005	0.005	0.005	0.005	0.005
Na_2_EDTA	0.08	0.04	0.04	0.04	0.04	0.04	0.04	0.04	0.04
K_2_SO_4_	1	0.5	0.5	0.5	0.5	0.5	0.5	0.5	0.5
MgSO_4_.7H_2_O	0.2	0.1	0.1	0.1	0.1	0.1	0.1	0.1	0.1
Trace element	1 mL	0.5 mL	0.5 mL	0.5 mL	0.5 mL	0.5 mL	0.5 mL	0.5 mL	0.5 mL

**Figure A1:**
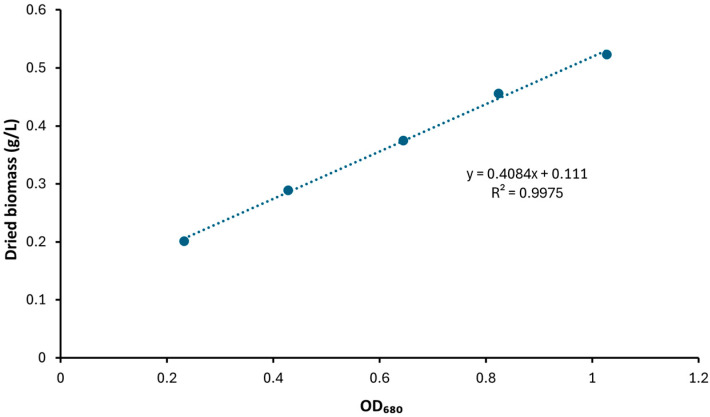
Calibration curve of optical density versus dried biomass of *Spirulina* sp. The blue dots represent the experimental data points, and the blue dotted line indicates the linear regression fit.

## Data Availability

The original contributions presented in this study are included in the article. Further inquiries can be directed to the corresponding author.

## Abbreviations

The following abbreviations are used in this manuscript:

3-PGA	3-phosphoglicerate
ALA	aminolevulinic acid
ANOVA	Analysis of Variance
BBM	Bold Basal Medium
BG-11	Blue Green-11
C	Control
CA	Carbonic anhydrase
C-PC	C-Phycocyanin
F1–F8	Formulations 1–8
GAP	glyceraldehide-3-phosphate
OD	Optical density
PCB	Phycocyanobilin
PCD	Phycocyanin content in dried biomass
PCL	Phycocyanin content in liquid
PI	Purity index
RuBisCO	Ribulose-1,5-bisphosphate carboxylase/oxygenase

## Appendix A

**Table A1:** Calibration curve data of optical density versus dry biomass of *Spirulina* sp.

	OD	Dried Biomass (g/L)
Replicate 1	1.000	0.572
	0.817	0.492
	0.621	0.390
	0.418	0.336
	0.223	0.234
Replicate 2	1.055	0.474
	0.831	0.420
	0.668	0.360
	0.439	0.242
	0.242	0.168
Average	1.027	0.523
	0.824	0.456
	0.644	0.375
	0.428	0.289
	0.232	0.201

Replicate 1 and Replicate 2 represent two independent calibration experiments relating optical density (OD_680_) to dry biomass of *Spirulina* sp., conducted under identical conditions to ensure reproducibility.
